# Bladder squamous cell cancer accompanied by Trousseau's syndrome: a case report

**DOI:** 10.1002/ccr3.1433

**Published:** 2018-02-21

**Authors:** Satoshi Kayukawa, Kenji Ina, Ryuichi Furuta, Tomoko Nishio, Tadayuki Miyashita, Shun Umeda, Takae Kataoka

**Affiliations:** ^1^ Department of Clinical Oncology Nagoya Memorial Hospital Nagoya Japan; ^2^ Department of Medical Oncology Nagoya Memorial Hospital Nagoya Japan; ^3^ Department of Pathology Nagoya Memorial Hospital Nagoya Japan; ^4^ Department of Neurology Nagoya Memorial Hospital Nagoya Japan; ^5^ Department of Urology Nagoya Memorial Hospital Nagoya Japan

**Keywords:** Bladder cancer, multiple cerebral infarctions, squamous cell carcinoma, Trousseau's syndrome

## Abstract

The association between thrombosis and cancer has been recognized since Trousseau's report in 1865. We present a case of bladder squamous cell carcinoma associated with multiple cerebral infarctions. This patient was diagnosed as having Trousseau's syndrome and received radiotherapy for bladder cancer treatment, along with anticoagulation therapy.

## Introduction

The association between neoplastic diseases and thromboembolic disorders is well recognized 1, 2. The incidence of thromboembolism in cancer patients is reported to be approximately 15% 3, despite the different rates for particular cancer sites and histological types 4. Since the first report in 1865 by Armand Trousseau 5, malignancy‐related thromboembolism has been referred to as Trousseau's syndrome, one of the paraneoplastic syndrome (PNS). PNS refers to symptoms or signs resulting from damage to organs or tissues that are remote from the site of a malignant neoplasm or its metastases. Approximately 10% of hospitalized cancer patients develop PNS with different clinical presentations including neurologic, endocrine, hematologic, rheumatologic, and dermatologic symptoms 6. PNS occurs most commonly in patients with small‐cell lung carcinoma, gynecological carcinomas, and lymphoma. However, bladder cancer has rarely been linked to a PNS such as Trousseau's syndrome 7. We herein describe a case of bladder squamous cell carcinoma accompanied by multiple cerebral infarctions due to an idiopathic thromboembolism.

## Case Presentation

**Figure 1:**
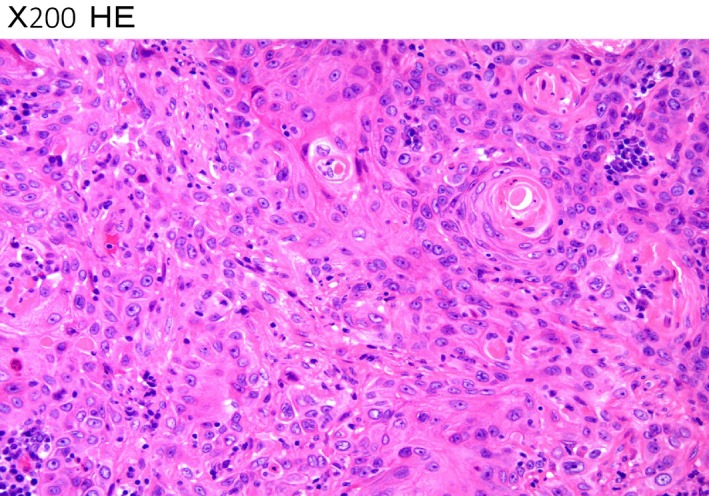
Microscopic examination of the bladder tumor by transurethral resection showed exclusively the squamous cell cancer without any other components of either adenocarcinoma or transitional cell carcinoma. HE, hematoxylin and eosin staining; x 200.

**Figure 2:**
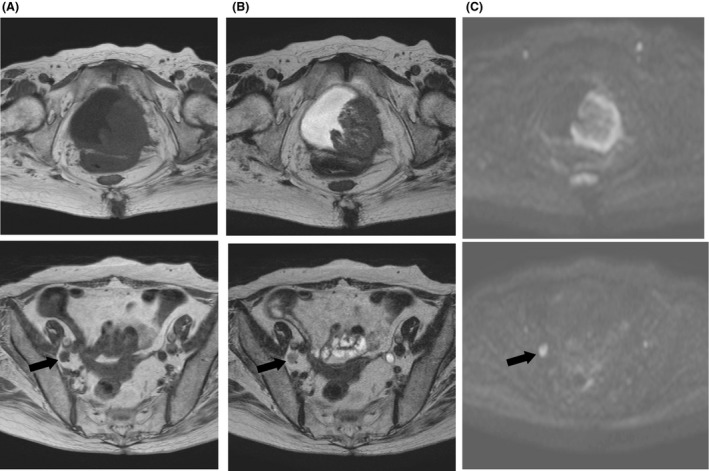
Magnetic resonance imaging. A huge mass (60 × 55 mm) in the bladder with lymph node enlargement (arrow) in the internal iliac region was observed. (A) T1‐weighted; (B) T2‐weighted (C) diffusion‐weighted.

**Figure 3:**
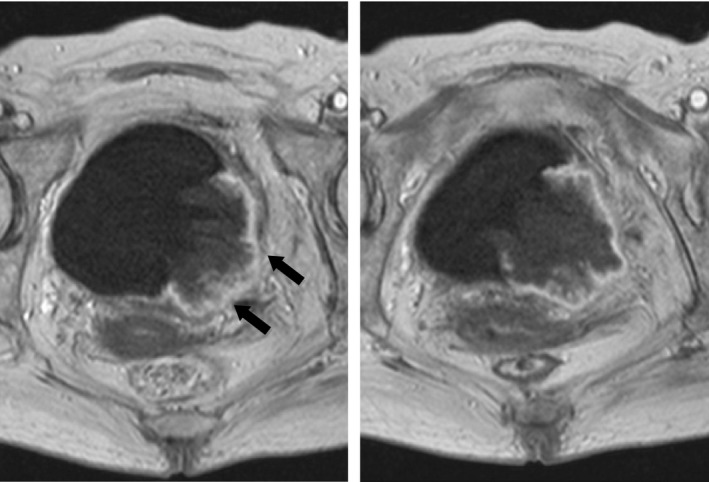
Contrast‐enhanced magnetic resonance imaging of the bladder revealed the interruption of muscle layer, and the depth of invasion was diagnosed as T2.

A 71‐year‐old woman was admitted for treatment of a 6‐cm bladder tumor. The initial symptoms included painless hematuria accompanied by loss of appetite. Transurethral resection of the bladder tumor (TURBT) was performed to make a histological diagnosis. Microscopic examination of the resected tumor showed exclusively the squamous cell cancer (Fig. 1), although the remained tumor might have the other components of either adenocarcinoma or transitional cell carcinoma. Staging evaluation using computed tomography (CT) and magnetic resonance imaging (MRI) revealed lymph node enlargement in the pelvic cavity (Fig. 2) without any evidence of distant metastasis such as liver, lung, and bone. Because the interruption of muscle layer was highly suspected by the findings of contrast‐enhanced MRI (Fig. 3), the patient was diagnosed with locally advanced disease (cT2 N1 M0) and platinum‐based chemotherapy was initiated prior to surgical resection. Informed consent for chemotherapy was obtained. Because the renal function was impaired due to the left‐sided hydronephrosis, 20 mg of cisplatin combined with 1000 mg of gemcitabine was administered. The patient suddenly complained of weakness in her right extremities on Day 5 after the start of chemotherapy.

**Figure 4:**
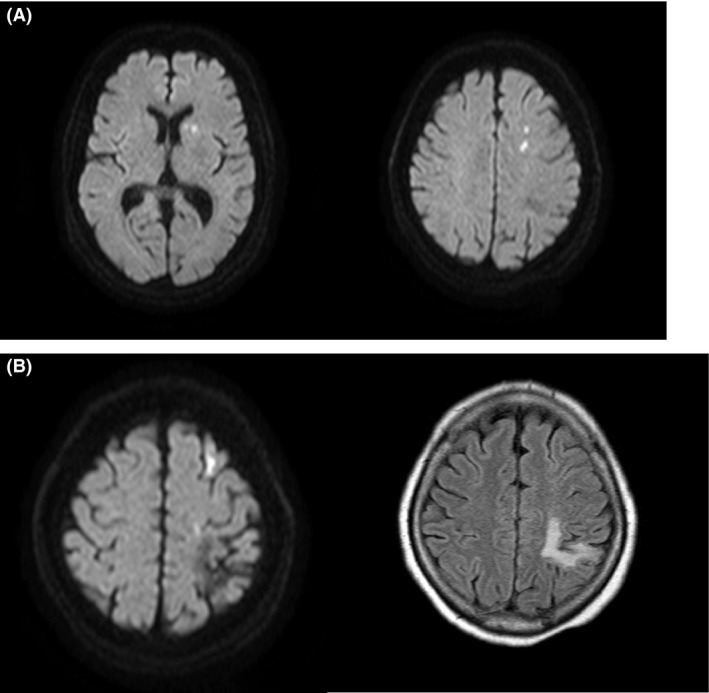
Magnetic resonance imaging sequences of the brain. Fresh multiple infarctions were observed in the cerebral white matter and basal ganglion as restricted diffusion (A), which were accompanied by an old infarction in the left parietal lobe (B).

On examination, the patient appeared well, with a regular pulse, blood pressure of 146/84 mmHg, normal heart sound, and no carotid bruits. A neurological examination revealed mild facial palsy and muscle weakness (biceps, 4/5; triceps, 4/5; lower limb, 4/5) on the right side. The blood coagulation test results on admission were as follows (normal range in parentheses): platelet count, 57.6 × 10^4^/*μ*L (14–34 × 10^4^/*μ*L); fibrinogen, 992 mg/dL (180–355 mg/dL); and D‐dimer, 0.4 *μ*g/mL (0–0.9 *μ*g/mL). A diffusion‐weighted MRI of the brain revealed multiple patchy shadows with a high signal intensity in the cerebral white matter and basal ganglia, indicating multiple fresh cerebral infarctions (Fig. 4A), without any occlusion of major arteries in the brain. An old infarction was also present in the left parietal lobe (Fig. 4B). There was no clinical or electrocardiographic evidence of an arrhythmia, and an echocardiogram revealed no evidence of a cardiac source for the emboli.

Glycerol infusion was initiated, and warfarin was administered with caution. The patient's right hemiplegia did not improve, even after the administration of anticoagulation treatment. Because the attack of cerebral infarction remarkably decreased her performance status (PS) to grade 3, cisplatin‐based chemotherapy could not be continued and then the radiation therapy (total dose, 60 Gy; 2 Gy/fraction) was initiated for the treatment of bladder tumor. After the radiation therapy, the impairment of renal function was sustained and her PS remained poor (PS 3), so we decided not to resume the chemotherapy. She eventually died of multiple liver metastases 8 months after the start of radiation without any recurrence of thromboembolic events.

## Discussion

Trousseau's syndrome was originally observed by Armand Trousseau 5, when he noted that some patients with unexpected or migratory thrombosis later presented with a visceral malignancy. This definition has been extended over the years, and the term “Trousseau's syndrome” currently refers to thrombotic events not explained by any obvious factors that occur concomitantly with either an occult or a recently diagnosed carcinoma 4. This is partly because even in its classic form, Trousseau's syndrome is probably mediated by multiple mechanisms including tissue factors 8, mucins 9, and oncogene activation 4. Although the precise mechanism remains uncertain, abnormal blood coagulation has been demonstrated in a subset of patients with malignancy. Our patient also had an abnormally high level of fibrinogen and elevated platelet count, which may be associated with thromboembolic diathesis. She presented with a neurological deficit on the right side during the first cycle of the combination chemotherapy with gemcitabine and cisplatin. In a retrospective analysis of 932 patients treated with cisplatin‐based chemotherapy, 169 (18.1%) experienced thromboembolic events 10. Although antineoplastic chemotherapy may have increased the risk of vascular events 11, it is reasonable to consider the cerebral infarctions being associated with an underlying malignancy in our patient based on the following reasons: First Figure 4B demonstrated that stroke of this patient preceded tumor diagnosis by several months, although she exhibited no neurological deficits at that time. In other words, she had already suffered from cerebrovascular events prior to the initiation of chemotherapy. Second, the fresh infarctions occurred very early in our patient, which differs from the analysis of chemotherapy‐induced thromboembolism describing that the median time until thromboembolic events occurrence was 48 days (interquartile range, 26–73 days) following administration of chemotherapy 10. Finally, our patient's infarctions were multiple, both in the cerebral white matter and basal ganglia. Among 271 patients receiving cisplatin for the treatment of urothelial carcinoma, three patients exhibited signs of cerebrovascular accidents and multiple cerebral infarctions were not entirely observed in this series of patients 12. These characteristics are more likely attributed to cancer‐associated thromboembolic diathesis rather than chemotherapy, and the vascular events could be considered as Trousseau's syndrome.

It has been reported that the risk of venous thromboembolism in malignancy varies according to the site of cancer and its lowest incidence is in patients with carcinoma of the bladder 13, 14. A literature search for cases of Trousseau's syndrome in association with bladder cancer was performed using the PubMed database. Trousseau's syndrome leading to multiple cerebral infarctions was first described in a 67‐year‐old man with transitional cell cancer of the bladder (Table 1) 15. He suffered from multiple infarctions in the occipital lobes bilaterally, with sudden loss of color vision. The second case is a 38‐year‐old woman, who experienced a sudden onset of visual field defects and lower limb weakness without any specific reasons 16. She was later diagnosed with bladder cancer accompanied by multiple bone metastases and enlarged lymph nodes. In contrast to the previous reports, our patient had been already diagnosed with squamous cell carcinoma of the bladder and then she developed cerebral infarctions, presenting with right hemiplegia, during the chemotherapy. Squamous cell carcinoma is an uncommon histological type of bladder cancer and is considered to be an aggressive tumor with a poor prognosis 17. Compared to the patients with squamous cell carcinoma, those with adenocarcinoma have a 1.65‐fold higher probability of developing an embolism (95% confidence interval: 1.20–2.29) 3. Experiments using animal models have demonstrated that a thrombogenic effect may be mediated by an interaction between mucin and selectin 18, which suggests that Trousseau's syndrome should be more frequently observed in mucin‐producing adenocarcinoma.

**Table 1:** Cases of bladder cancer accompanied by multiple cerebral infarctions

Age	Gender	Symptoms	Site	Histological type	Treatment	Outcome
67	Male	Achromatopsia	Bilateral occipital lobe	Transitional cell carcinoma	Heparin	Dead (3 months)
38	Female	Visual field defects Lower limb weakness	Bilateral cerebral white matter	Transitional cell carcinoma	Heparin	Dead (3 months)
71	Female	Right hemiplegia	Left cerebral white matter and basal ganglion	Squamous cell carcinoma	Radiotherapy Warfarin	Dead (8 months)

Despite these underlying mechanisms, the primary approach to treating Trousseau's syndrome is to eliminate the causative tumor. In the present case, chemotherapy could not be resumed due to the decreased PS and sustained renal impairment. Radiotherapy was initiated aiming at reducing the tumor burden, as squamous cell cancer is known to be sensitive to radiation 17. Concomitant warfarin anticoagulation treatment was administered. Trousseau's syndrome has been reported to be associated with a high mortality rate 3, 16. Our patient also died within a year, although she did not have any distant metastasis at the time of diagnosis.

In conclusion, Trousseau's syndrome rarely develops in patients with cancers originating in the bladder. The use of anticoagulation, along with radiation therapy, successfully controlled the thromboembolic events in the patient with squamous cell carcinoma of the bladder.

## Consent

Written informed consent was obtained from the patient for publication of this case report and accompanying images. A copy of the written consent is available for review by the editor of this journal.

## Authorship

SK and KI: equally contributed to writing the manuscript. SK, RF, and SU: interpreted the radiologic examination findings. TN: performed the histological examination of the bladder tumor and diagnosed the pathological findings. TM: made the neurological diagnosis. TK: reviewed the manuscript. All authors read and approved the final manuscript.

## Conflict of Interest

The authors declare that they have no competing interests.
